# Schmallenberg Virus Infection of Adult Type I Interferon Receptor Knock-Out Mice

**DOI:** 10.1371/journal.pone.0040380

**Published:** 2012-07-06

**Authors:** Kerstin Wernike, Angele Breithaupt, Markus Keller, Bernd Hoffmann, Martin Beer, Michael Eschbaumer

**Affiliations:** 1 Institute of Diagnostic Virology, Friedrich-Loeffler-Institut (FLI), Greifswald–Insel Riems, Germany; 2 Department of Experimental Animal Facilities and Biorisk Management, Friedrich-Loeffler-Institut (FLI), Greifswald–Insel Riems, Germany; 3 Institute of Novel and Emerging Infectious Diseases, Friedrich-Loeffler-Institut (FLI), Greifswald–Insel Riems, Germany; Auburn University, United States of America

## Abstract

Schmallenberg virus (SBV), a novel orthobunyavirus, was discovered in Europe in late 2011. It causes mild and transient disease in adult ruminants, but fetal infection can lead to abortion or severe malformations. There is considerable demand for SBV research, but *in vivo* studies in large animals are complicated by their long gestation periods and the cost of high containment housing. The goal of this study was to investigate whether type I interferon receptor knock-out (IFNAR^−/−^) mice are a suitable small animal model for SBV. Twenty IFNAR^−/−^ mice were inoculated with SBV, four were kept as controls. After inoculation, all were observed and weighed daily; two mice per day were sacrificed and blood, brain, lungs, liver, spleen, and intestine were harvested. All but one inoculated mouse lost weight, and two mice died spontaneously at the end of the first week, while another two had to be euthanized. Real-time RT-PCR detected large amounts of SBV RNA in all dead or sick mice; the controls were healthy and PCR-negative. IFNAR^−/−^ mice are susceptible to SBV infection and can develop fatal disease, making them a handy and versatile tool for SBV vaccine research.

## Introduction

In late 2011, Schmallenberg virus (SBV), an orthobunyavirus that is related to Akabane virus (AKAV), was first identified in cattle in Europe [1]. It is associated with mild transient disease in adult cattle, but can cause severe fetal malformations (arthrogryposis-hydranencephaly syndrome) when pregnant cows and ewes are infected in early to mid pregnancy. Currently, very little is known about SBV; substantial efforts will be required to elucidate its pathogenesis and develop preventive strategies.

Research on ruminant viruses is complicated by the high costs for large animals and adequate high containment housing, but this can be mitigated by suitable small animal models. In recent years, research on orbiviruses, another group of arboviruses of livestock, has benefitted greatly from the introduction of type I interferon receptor knock-out mice (IFNAR^−/−^) [2]–[4]; accordingly, this study investigated the utility of IFNAR^−/−^ mice for SBV.

## Materials and Methods

### Mice

Twenty-four IFNAR^−/−^ mice on a C57BL/6 genetic background were obtained from the specific-pathogen-free breeding unit of the FLI. The average age of the mice on the day of inoculation was 8.4±0.4 weeks; mice of both sexes were distributed evenly among the groups.

The experimental protocol was reviewed by an independent ethics commission pursuant to §15 of the German Animal Welfare Act, and it has been approved by the competent authority (State Office for Agriculture, Food Safety and Fisheries of Mecklenburg-Vorpommern, Rostock, Germany, ref. LALLF M-V TSD/7221.3-1.1-004/12).

### Virus

Schmallenberg virus was isolated from the blood of an infected cow as previously described [1]. After isolation on KC cells (derived from *Culicoides variipennis* midges [5]), the virus was passaged once in baby hamster kidney (BHK) cells and again in KC (Cell lines L179 and L1062, respectively; both are available from the Collection of Cell Lines in Veterinary Medicine of the Friedrich-Loeffler-Institut). The infectivity of the KC lysate was determined by end-point titration on BHK cells.

Twenty IFNAR^−/−^ mice were inoculated subcutaneously into the scruff of the neck with 0.8×10̂3 50% tissue culture infectious doses of SBV. Four mice were injected with phosphate-buffered saline and kept as controls. All mice were weighed and examined daily for the duration of the experiment.

### Real-time RT-PCR

Beginning on day 1 after infection, two mice per day were euthanized by exsanguination under anesthesia and samples were collected from their blood, brain, lungs, liver, spleen and small intestine. Three mice were sampled on day 7 (see below); therefore only one inoculated mouse remained at the end of the experiment on day 10. The control mice were also euthanized and sampled on that day.

Collected samples were homogenized in a TissueLyser (Qiagen, Hilden, Germany); after centrifugation, RNA was extracted from the supernatants with the NucleoSpin® 96 RNA Kit (Macherey-Nagel, Düren, Germany) on a MICROLAB® STAR liquid handling workstation (Hamilton, Bonaduz, Switzerland). All samples were tested in an SBV-specific real-time RT-PCR [6] with an external standard based on the S genome segment.

### Pathology

Representative samples were submitted for histopathological investigation. The liver of mouse 5a (euthanized on day 5, first animal with macroscopic changes) and the small intestine of mouse 7b (found dead on day 7 with profuse abdominal bleeding; see below) were fixed in 4% phosphate-buffered neutral formaldehyde and embedded in paraffin. Sections (4 µm) were cut and stained with hematoxylin and eosin (HE). Immunostaining was not possible, because no SBV-specific antibody suitable for formalin-fixed, paraffin-embedded tissue was available.

## Results

### Clinical disease and pathology

**Figure 1:**
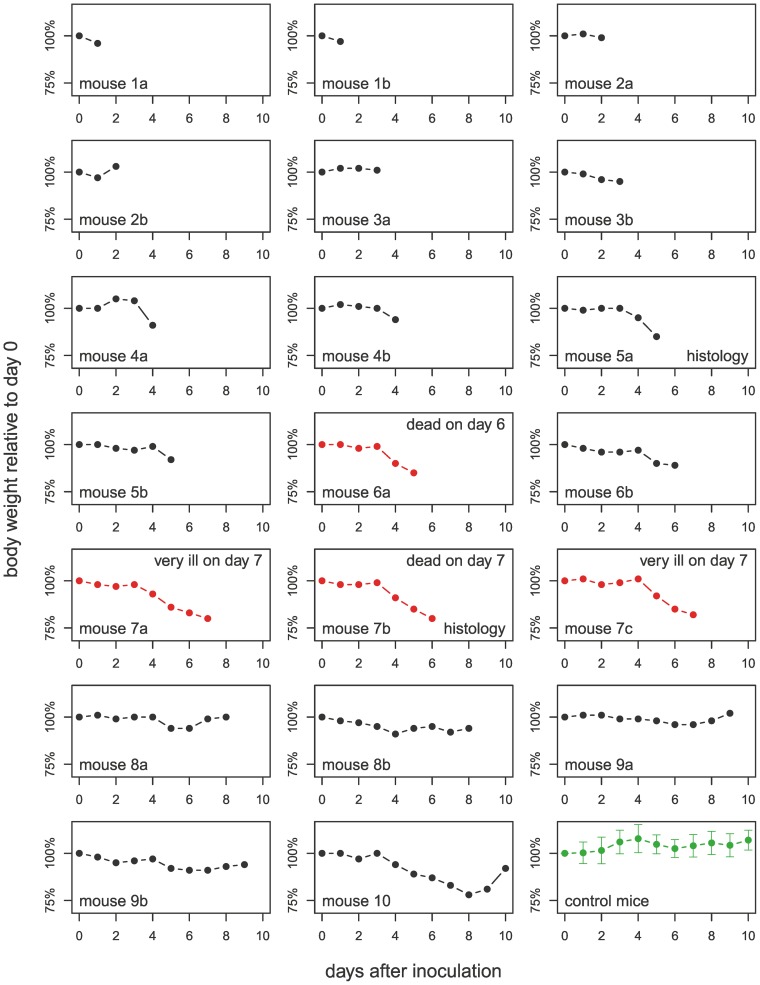
Body weights of mice. Mice marked in red died spontaneously or had to be sacrificed prematurely; mice 7a and 7c were euthanized on day 7. Tissues of mice 5a and 7b were histologically examined, see figures 3 and 4.

From day 4 onwards, inoculated mice started to lose weight (see figure 1). Two mice were found dead on days 6 and 7 (mouse 6a and 7b, respectively); on day 7, two more (7a and 7c) had to be euthanized for humane reasons because they appeared severely ill and were unresponsive.

**Figure 2:**
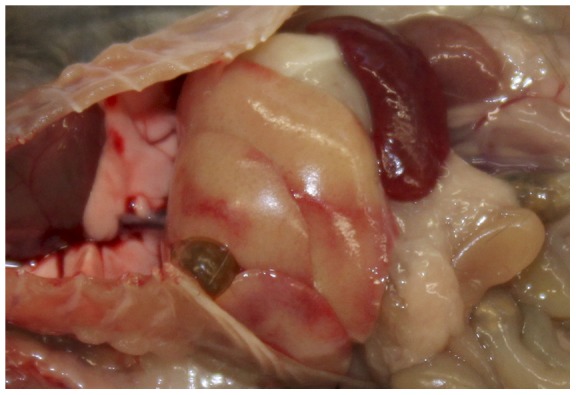
Gross pathology, abdominal cavity, mouse 5a. Enlarged spleen and diffusely beige liver with multifocal hemorrhages.

**Figure 3:**
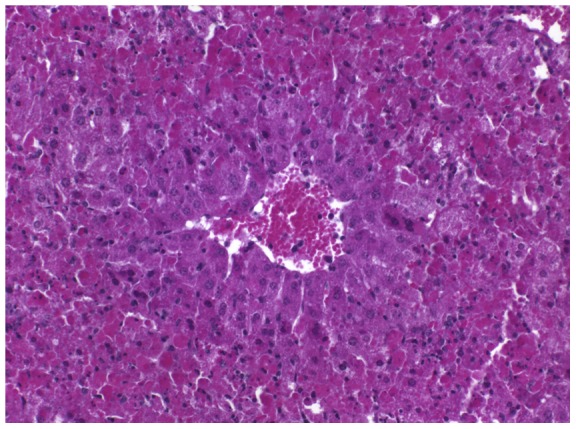
Histopathology, liver, mouse 5a. HE stain. Hepatocellular centroacinar degeneration (periportal, zone 1) and necrosis.

**Figure 4:**
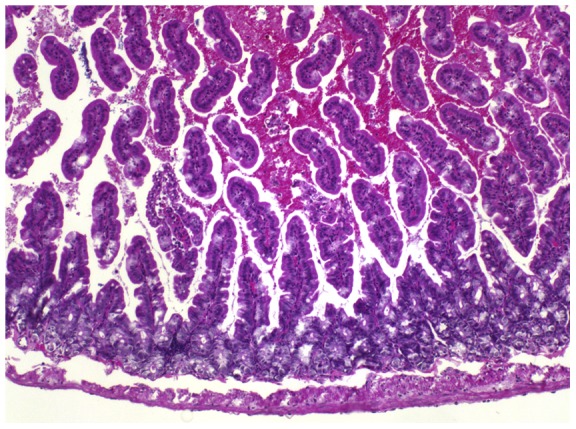
Histopathology, small intestine, mouse 7a. HE stain. Intestinal lumen filled with abundant erythrocytes and scattered exfoliated enterocytes.

At necropsy, the livers of several mice appeared diffusely discolored to beige (see figure 2 for an example); histomorphologically, severe hepatocellular degeneration and necrosis were evident (mouse 5a; figure 3). The abdominal cavities of mice 6a and particularly 7b contained large amounts of blood; internal bleeding into the small intestine was also confirmed by histopathology (mouse 7b, figure 4).

### Real-time RT-PCR

**Figure 5:**
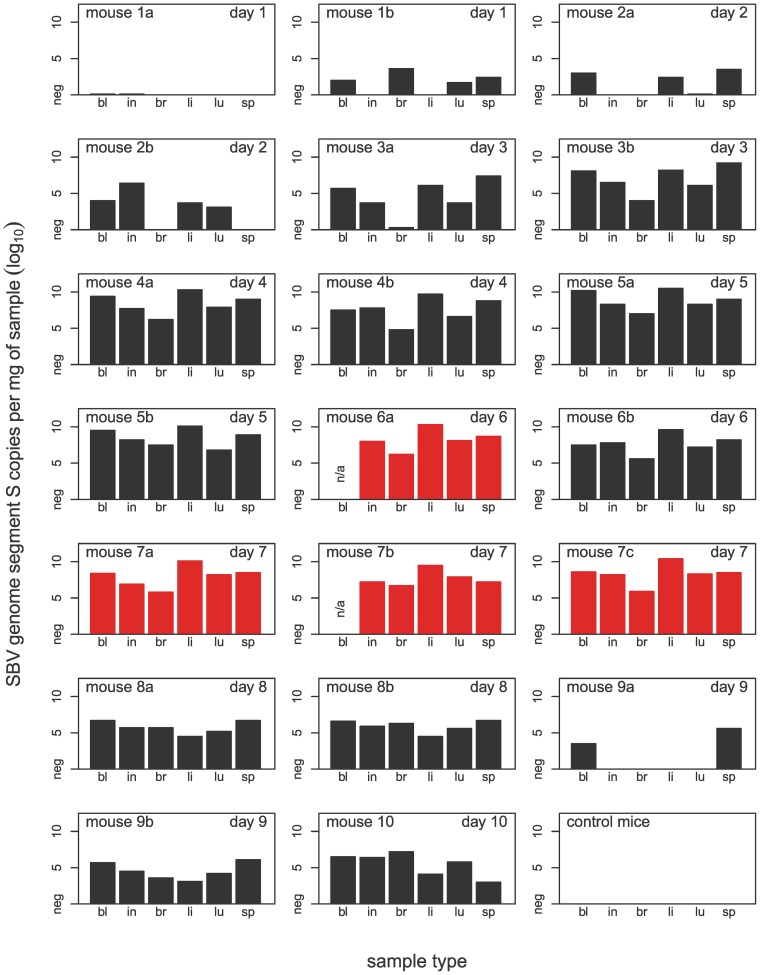
Real-time RT-PCR results for blood, intestine, brain, liver, lungs, and spleen. Mice marked in red died spontaneously or had to be euthanized prematurely; see figure 1 for details. Since mice 6a and 7b were already dead, no blood could be collected.

Samples taken from dead and euthanized mice were highly positive in the PCR (lowest quantification cycle [Cq] value in a liver sample: 10.7, corresponding to 2.3×10̂10 S-segment copies per mg of tissue). Substantial amounts of SBV RNA were also found in the other tissues of mice that had been losing weight; it was much less abundant in inconspicuous mice (no weight loss; see figure 5). All controls were negative.

## Discussion

Owing to its recent discovery, there is no literature on SBV. Its close relation to AKAV [1], however, suggests that much of what is known about that virus might also be applicable here. AKAV isolation and propagation in mice was first described over 30 years ago [7], but to this day it requires the intracranial injection of newborns [8]. Obviously, this route of inoculation is very arduous, and newborn mice cannot be used for vaccination experiments. Despite their compromised innate immune response, vaccination can protect adult IFNAR^−/−^ mice against fatal viral infection [9]; they have been used for the evaluation of prototype vaccines against a variety of viruses [10]–[14], including La Crosse virus, another orthobunyavirus [15].

In the present experiment, subcutaneous inoculation of adult IFNAR^−/−^ mice with SBV led to disease manifested by weight loss and, in severe cases, apathy and death. Hemorrhagic diathesis was evident and is suspected as the cause of death in at least two mice. Hemorrhages occur through abnormal function of blood vessels (e.g. endothelial damage), platelets (reduced production or dysfunction) or coagulation factors (disturbed clotting cascade, consumption of clotting factors). The liver damage suggests an intrahepatic consumption of clotting factors due to endothelial necrosis and potentially a failure to produce clotting factors. The pathogenesis of SBV-induced hemorrhagic diathesis needs to be examined in greater detail; similar observations have been reported for IFNAR^−/−^ mice infected with Crimean-Congo hemorrhagic fever virus [16].

The extent of weight loss before euthanasia generally corresponded with the severity of lesions discovered at necropsy and the amount of SBV RNA in tissues. SBV RNA was found in all examined tissues of all animals euthanized from day 3 onwards. One mouse (9a, euthanized on day 9) was only positive in the blood and spleen; this was also the animal that had displayed the least degree of weight loss. Despite losing a lot of weight between days 3 and 8, mouse 10 was already recovering quickly before it was euthanized at the end of the experiment on day 10; nevertheless, a systemic SBV infection was demonstrated by real-time RT-PCR. When suitable antibodies become available, this can be confirmed by immunostaining of tissue sections. Further studies will be necessary to determine whether SBV infection of IFNAR^−/−^ mice is dose-dependent, as is indeed suggested by the disparate clinical outcome in the present experiment.

As shown in this experiment, IFNAR^−/−^ mice are susceptible to SBV infection and can develop fatal disease, making them a handy and versatile tool for SBV vaccine research. If transplacental transmission in pregnant mice can be demonstrated, they could also be used to elucidate the pathogenesis of arthrogryposis-hydranencephaly syndrome; apart from the reduced cost and effort offered by a small animal model, the considerably shorter gestation period of mice is particularly beneficial for *in vivo* studies of fetal SBV infection.

## References

[1] Hoffmann B, Scheuch M, Höper D, Jungblut R, Holsteg M (2012). Novel orthobunyavirus in cattle, Europe, 2011.. Emerg Infect Dis.

[2] Calvo-Pinilla E, Rodríguez-Calvo T, Anguita J, Sevilla N, Ortego J (2009). Establishment of a bluetongue virus infection model in mice that are deficient in the alpha/beta interferon receptor.. PLoS One.

[3] Eschbaumer M, Keller M, Beer M, Hoffmann B (2012). Epizootic hemorrhagic disease virus infection of type I interferon receptor deficient mice.. Vet Microbiol.

[4] Castillo-Olivares J, Calvo-Pinilla E, Casanova I, Bachanek-Bankowska K, Chiam R (2011). A modified vaccinia Ankara virus (MVA) vaccine expressing African horse sickness virus (AHSV) VP2 protects against AHSV challenge in an IFNAR −/− mouse model.. PLoS One.

[5] Wechsler SJ, McHolland LE, Wilson WC (1991). A RNA virus in cells from Culicoides variipennis.. J Invertebr Pathol.

[6] Bilk S, Schulze C, Fischer M, Beer M, Hlinak A (2012). Organ distribution of Schmallenberg virus RNA in malformed newborns.. Vet Microbiol, in press.

[7] Kurogi H, Inaba Y, Takahashi E, Sato K, Omori T (1976). Epizootic congenital arthrogryposis-hydranencephaly syndrome in cattle: isolation of Akabane virus from affected fetuses.. Arch Virol.

[8] Oem JK, Yoon HJ, Kim HR, Roh IS, Lee KH (2012). Genetic and pathogenic characterization of Akabane viruses isolated from cattle with encephalomyelitis in Korea.. Vet Microbiol, in press.

[9] Müller U, Steinhoff U, Reis LF, Hemmi S, Pavlovic J (1994). Functional role of type I and type II interferons in antiviral defense.. Science.

[10] Franceschi V, Capocefalo A, Calvo-Pinilla E, Redaelli M, Mucignat-Caretta C (2011). Immunization of knock-out alpha/beta interferon receptor mice against lethal bluetongue infection with a BoHV-4-based vector expressing BTV-8 VP2 antigen.. Vaccine.

[11] Calvo-Pinilla E, Rodríguez-Calvo T, Sevilla N, Ortego J (2009). Heterologous prime boost vaccination with DNA and recombinant modified vaccinia virus Ankara protects IFNAR(−/−) mice against lethal bluetongue infection.. Vaccine.

[12] Ma G, Eschbaumer M, Said A, Hoffmann B, Beer M (2012). An equine herpesvirus type 1 (EHV-1) expressing VP2 and VP5 of serotype 8 bluetongue virus (BTV-8) induces protection in a murine model of infection.. PLoS One.

[13] Lorenzo G, Martin-Folgar R, Hevia E, Boshra H, Brun A (2010). Protection against lethal Rift Valley fever virus (RVFV) infection in transgenic IFNAR(−/−) mice induced by different DNA vaccination regimens.. Vaccine.

[14] Operschall E, Schuh T, Heinzerling L, Pavlovic J, Moelling K (1999). Enhanced protection against viral infection by co-administration of plasmid DNA coding for viral antigen and cytokines in mice.. J Clin Virol.

[15] Schuh T, Schultz J, Moelling K, Pavlovic J (1999). DNA-based vaccine against La Crosse virus: protective immune response mediated by neutralizing antibodies and CD4+ T cells.. Hum Gene Ther.

[16] Bereczky S, Lindegren G, Karlberg H, Akerstrom S, Klingstrom J (2010). Crimean-Congo hemorrhagic fever virus infection is lethal for adult type I interferon receptor-knockout mice.. J Gen Virol.

